# Beyond Drug Reward: Heritable Sensitivity to Cocaine Aversion as a Determinant of Addiction Vulnerability

**DOI:** 10.1523/ENEURO.0163-26.2026

**Published:** 2026-06-05

**Authors:** Ginevra D’Ottavio, Yavin Shaham

**Affiliations:** Intramural Research Program, NIDA, NIH, Baltimore, Maryland 21224

Epidemiological studies show that only a minority of people exposed to addictive drugs develop addiction, suggesting that preexisting biological factors contribute to addiction vulnerability. Genome-wide association studies support this hypothesis by identifying genetic variants that are partially shared across different addictive drugs (Lai et al., 2026). Twin and family studies similarly indicate that addiction-related traits are heritable. For example, unaffected siblings of people with stimulant addiction show behavioral and neurobiological traits associated with addiction vulnerability, including impulsivity and impaired inhibitory control; these results suggest that inherited factors contribute to addiction risk even before drug exposure (Ersche et al., 2012). Although environmental influences and life experiences also contribute to addiction development, the biological mechanisms underlying vulnerability and resilience remain incompletely understood.

One hypothesis is that individuals who develop addiction are biologically more sensitive to the rewarding effects of drugs. Human studies support this idea by showing that stronger stimulant and rewarding responses during initial drug exposure predict later drug use and addiction. For example, greater alcohol-induced stimulation, liking, and wanting in recreational drinkers predict future heavy alcohol use, and these positive subjective effects progressively increase in people who later develop alcohol addiction (King et al., 2025). Similarly, genetic polymorphisms can influence subjective drug responses. Variants of the μ-opioid receptor gene (OPRM1), including the A118G polymorphism, alter receptor signaling and are associated with differences in the subjective and reinforcing effects of opioids (Dunn et al., 2024).

However, addiction vulnerability may depend not only on sensitivity to rewarding drug effects but also on sensitivity to aversive drug effects. Different addictive drugs produce negative subjective effects during initial exposure, including dysphoria, nausea, sedation, and conditioned aversion, which can influence subsequent drug use. Thus, heightened sensitivity to aversive drug effects may represent an inherited mechanism of resilience to addiction.

Evidence for this idea comes from studies using selectively bred alcohol-preferring (P) rats. Across generations of selective breeding, these rats show reduced sensitivity to the aversive, ataxic, and hypothermic effects of alcohol compared with alcohol-nonpreferring (NP) rats while consuming large amounts of alcohol (Bell et al., 2006). These findings suggest that reduced sensitivity to alcohol's aversive effects increases vulnerability to alcohol addiction, whereas greater sensitivity may promote resilience. Like alcohol, cocaine produces both rewarding and aversive effects, and both people and lab animals differ in their sensitivity to these effects. However, over the last several decades, the role of cocaine's aversive effects in addiction has received relatively little attention in both human and animal research. One reason is that cocaine is a potent operant reinforcer that reliably supports self-administration in mice, rats, monkeys, and humans. In addition, standard operant self-administration procedures are not well suited for studying drug aversion.

Cocaine aversive effects can be studied using the runway model and conditioned place preference/aversion (CPP/CPA) procedures (Ettenberg, 2009; Su et al., 2013). In the runway model, rodents traverse an alley to a goal box where they receive an appetitive stimulus such as drug infusions or palatable food or an aversive stimulus, such as footshock. In the CPP procedure, a reinforcer is paired with a specific context, while a different context is paired with nonreinforcement, and preference for the reinforcer-paired context is later assessed in the absence of the reinforcer. For example, when palatable food is used as the reinforcer, rats progressively decrease their runtime in the runway and develop CPP for the food-paired compartment. In contrast, footshock increases runtime and produces CPA.

Beginning in the 1980s, Ettenberg and colleagues used the runway and CPP/CPA procedures to study cocaine's rewarding and aversive effects (Ettenberg, 2009; Su et al., 2013). Two major findings emerged. First, when intravenous cocaine served as the reinforcer in the goal box, runway runtime paradoxically increased over sessions, resembling the response to shock rather than food or heroin. Rats often show conflict-like behavior characterized by repeated advances and retreats before entering the goal box, similar to the behavior observed when food and shock are simultaneously associated with the goal box. Second, in the CPP/CPA procedure, rats developed CPP when cocaine was administered immediately before context exposure but developed CPA when cocaine was administered 15 min before context exposure.

Together, the studies of Ettenberg and colleagues established experimental procedures for studying cocaine aversion and identified substantial individual variability in aversive responses to cocaine. However, whether these individual differences reflect heritable traits is unknown.

In a recent study published in eNeuro, the Jhou lab (Eid et al., 2026) addressed this question using the runway and CPP/CPA procedures in heterogeneous stock (HS) rats and several additional strains, including outbred Sprague-Dawley rats and the inbred Agouti, Brown Norway, Buffalo, Fischer, Lewis, Maudsley Non-Reactive (M520/N), and Wistar Kyoto strains. HS rats are a genetically diverse outbred population generated by intercrossing eight inbred founder strains [August × Copenhagen Irish (ACI/N), Brown Norway (BN/SsN), Buffalo (BUF/N), Fischer 344 (F344/N), Maudsley Reactive (MR/N), Maudsley Non-Reactive (M520/N), Wistar Kyoto (WKY/N), and Wistar (WN/N)], followed by many generations of breeding.

The main finding of the study was that heritable factors strongly influence cocaine aversion. The authors showed that offspring from two generations of selectively bred outbred Sprague Dawley and HS rats with high or low cocaine avoidance in the runway task and also showed high or low cocaine avoidance in both the runway and CPA tasks. The estimated heritability (h²) for cocaine avoidance in the first generation was *h*² = 0.49 in outbred Sprague Dawley rats and *h*² *=* 0.63 in the HS rats. These values, which are within the moderate to high heritability range, were independent of sex. Large strain differences were also observed, with some strains showing low cocaine avoidance (Lewis and Brown Norway) and others showing high avoidance (Buffalo), further supporting a heritable contribution to cocaine aversion.

Importantly, control experiments showed that individual differences in cocaine avoidance could not be explained by variation in exploratory locomotion or general motivation, as measured by progressive-ratio responding for food reward. Resistance to punishment, a trait linked to addiction-like behavior, differed across strains but was unrelated to cocaine avoidance. These findings suggest that cocaine avoidance and punishment resistance are mediated by distinct heritable factors. Finally, rats with high and low cocaine avoidance showed similar cocaine CPP when cocaine was administered immediately before context exposure, suggesting dissociable genetic susceptibility to cocaine reward versus aversion.

At the neurobiological level, the authors confirmed previous findings from Jhou and colleagues (Chao et al., 2023) showing that greater cocaine avoidance is associated with increased Fos expression, a marker of neuronal activity, in the rostromedial tegmental nucleus (RMTg) following cocaine infusion. This finding suggests that activity within this region represents a neural marker of cocaine aversion.

Together, the authors' findings provide converging evidence that cocaine aversion, as measured in the runway and CPA procedures, is a heritable trait that is independent of motivation for food reward, resistance to punishment, and acute cocaine reward measured by CPP. An important unanswered question is whether individual differences in cocaine aversion predict addiction vulnerability in self-administration-based models.

In this regard, the Jhou lab previously showed that rats with high cocaine aversion acquired cocaine self-administration more slowly. With additional training, these rats eventually reached levels of self-administration comparable to those with low-aversion rats, suggesting that drug exposure and other non-heritable factors also contribute to acquisition of cocaine self-administration (Chao et al., 2023). However, after acquisition, high-aversion rats showed faster extinction of the cocaine-reinforced responding and lower cue-induced reinstatement after extinction, consistent with a persistent protection against addiction-like behaviors.

Future preclinical studies should determine whether low- and high-aversion phenotypes predict addiction-like behavior in more formal models of addiction (Venniro et al., 2020), including DSM-IV-based models, extended-access and intermittent-access procedures, and a more recent model in which “addiction-vulnerable” rats choose drug (heroin) over rewarding social interaction (D’Ottavio et al., 2025).

More importantly, at the clinical level, the present findings and the seminal work of Ettenberg and colleagues should encourage researchers to incorporate measures of cocaine aversion into longitudinal studies of addiction trajectories, similar to recent work examining subjective alcohol responses in humans (King et al., 2025).

Finally, from a medication-development perspective, studies of cocaine aversion suggest that experimental manipulations targeting brain regions involved in cocaine aversion, such as the RMTg or the lateral habenula (Chao et al., 2023; Gomez et al., 2025), may help counteract cocaine reward and complement behavioral and pharmacological treatments such as contingency management or agonist replacement therapies that reduce cocaine misuse.

## Synthesis

Reviewing Editor: Mihaela Iordanova, Concordia University - Loyola Campus Decisions are customarily a result of the Reviewing Editor and the peer reviewers coming together and discussing their recommendations until a consensus is reached. When revisions are invited, a fact-based synthesis statement explaining their decision and outlining what is needed to prepare a revision will be listed below. The following reviewer(s) agreed to reveal their identity: NONE.

Dear Ginevra and Yavin,

Than you for your excellent commentary on Eid et al. I enjoyed reading it and was delighted to learn more about cocaine aversion.

Mihaela
